# Long-term plasticity of astrocytic phenotypes and their control by neurons in health and disease

**DOI:** 10.1042/EBC20220090

**Published:** 2023-03-03

**Authors:** Kyle S. Wardlaw, Giles E. Hardingham

**Affiliations:** 1 UK Dementia Research Institute at The University of Edinburgh, Edinburgh Medical School, Edinburgh EH16 4TJ, U.K.; 2 Centre for Discovery Brain Sciences, Edinburgh Medical School, University of Edinburgh, Edinburgh EH8 9XD, U.K.

**Keywords:** astrocytes, homeostasis, transcription

## Abstract

The brain is a complex organ even when viewed from a cell biological perspective. Neuronal networks are embedded in a dense milieu of diverse and specialised cell types, including several types of vascular, immune, and macroglial cells. To view each cell as a small cog in a highly complex machine is itself an oversimplification. Not only are they functionally coupled to enable the brain to operate, each cell type’s functions are themselves influenced by each other, in development, maturity, and also in disease. Astrocytes are a type of macroglia that occupy a significant fraction of the human forebrain. They play a critical role in sustaining functional neuronal circuits across the lifespan through myriad homeostatic functions including the maintenance of redox balance, ionic gradients, neurotransmitter clearance, and bioenergetic support. It is becoming apparent that astrocytes’ capacity to carry out these and other neurosupportive roles is not fixed, but is regulated by signals coming from the neurons themselves, both in the healthy brain but also in response to neuron-derived disease pathology. Here, we review mechanisms by which neurons control the properties of astrocytes long term in order to alter their homeostatic capacity both in development and maturity. Our working hypothesis is that these signals are designed to change and maintain the homeostatic capacity of local astrocytes to suit the needs of nearby neurons. Knowledge of the external signals that can control core aspects of a healthy astrocytic phenotype are being uncovered, raising the question as to whether this knowledge can be harnessed to promote astrocyte-mediated neurosupport in brain disorders.

## Introduction

Glia, immune cells, and the vasculature all play key roles in maintaining functional postmitotic neuronal circuits over long periods of time (several decades in the case of humans). Astrocytes are a major type of glial cells that possess specialised functions and morphology that are essential for neuro- and synapto-protection in the healthy brain [1–3]. Such neurosupportive functions are diverse, with many metabolic pathways *in vivo* spanning both neurons and astrocytes, such as those associated with neurotransmitter metabolism, ion homeostasis, or bioenergetic coupling [1,4]. These and other pathways involve steps that are totally dependent on one of the cell types, and the transport of metabolites and other molecules from one cell to the other [1–3]. This intimate relationship thus requires close co-operation and the capacity of neurons and astrocytes to execute their role in each pathway to be well-balanced. As neurons and astrocytes cell fates are specified during development they acquire many of these specialised functions as part of the differentiation process [5].

A healthy astrocyte is constantly responding to neuronal signals to resolve homeostatic challenges on the millisecond to minute scale. Due to rapid uptake capacity, neuronal release of neurotransmitters and ions such as potassium (K^+^) can be cleared at the subsecond scale. However, neurons respond rapidly and post-translationally to neuron-derived signals. For example, neuronally released K^+^, nitric oxide (NO), and ammonium induce intracellular pathways to rapidly modulate astrocytic metabolism, including enhanced astrocytic glucose uptake, suppress oxidative phosphorylation, and enhance lactate export [6]. These and other acute responses to neuronally-derived signals (arguably a form of short-term plasticity) are reviewed comprehensively elsewhere [1,6,7].

However, the long-term phenotype of properties of astrocytes are also not fixed. There is growing evidence that the capacity of astrocytes to carry out several specialised functions is subject to signal-dependent regulation by neurons. The teleological reason for this is appears to be straightforward: to tune the astrocytes’ properties to the needs of nearby neurons, and enable them to effectively respond in the dynamic way touched on above [1,6,7]. The article will give examples of this mode of regulation as well the mechanisms involved. Since many astrocytic neurosupportive functions decline in age and disease [8–10], knowledge of how they are regulated in the healthy brain may point to ways in which they can be manipulated for therapeutic effect.

## Regulation of astrocyte morphology

It has long been known that astrocytes maintained in a monoculture have a simple nonramified morphology and that upon coculture with neurons acquire their characteristic stellate shape [5,11]. This strongly suggested that a CNS-derived signal was required to induce and maintain this aspect of astrocytes. The stellate morphology of astrocytes *in vivo* is important functionally as they must have fine protrusions to be able to contact the synaptic cleft and contribute to both neurotransmitter and ionic homeostasis there [12]. Additionally they have active functions via these neuroproximal processes, contributing to neuromodulatory signalling to control neuronal activity, impacting on circuit function and ultimately behaviour [13].

Recently, a juxtacrine signalling pathway involving the interaction between astrocytic neuroligin-2 and neuronal neurexins was shown to be a major contributor to the neuron coculture-induced morphological complexity of astrocytes [14], and that neuroligins 2 and 3 contributed to morphological development *in vivo* [14]. Interestingly, loss of astrocytic neuroligin-2 also resulted in a reduction in excitatory synapses at the structural and functional level in the developing brain. It remains to be seen whether the morphological changes in astrocytes driven by neuroligin–neurexin interactions are mechanistically linked to excitatory synapse number or whether they are two parallel neuroligin-dependent processes. In the future, it will be of interest to know whether astrocyte–neuron neuroligin–neurexin interactions, and the downstream effects on astrocyte morphology are themselves subject to regulation. Recently, *trans*-synaptic neuroligin–neurexin interactions have been shown to be controlled by astrocyte-secreted proteins SPARC and SPARCL1 [15], raising the possibility that similar astrocyte–neuron interactions can also be controlled.

The control of astrocytic morphology also bears relevance to disease. For example, astrocyte morphology changes in Alzheimer’s disease, albeit with strong regional variability and heterogeneity [16], although it appears that in vulnerable regions there is a reduction in coverage by perisynaptic processes which, coupled with a reduction in neurotransmitter uptake capacity [17] (and see below) would be predicted to have a strong effect on the fidelity of synaptic transmission. Another important translational aspect of astrocyte morphology is the difference between rodent and human astrocytes, the latter of which are far larger and more complex, even when grafted into rodents [18], and possess different levels of heterogeneity in health (possessing primate-specific subtypes) and also disease [16].

## Regulation of neurotransmitter uptake

Neurotransmitter uptake following presynaptic release plays an important role in shaping the postsynaptic response, in terms of response kinetics, the frequency-dependent integration of presynaptic bursts, as well as determining the extent of spillover from the synaptic cleft and activation of extrasynaptic receptors, activation of which can be toxic [19–21]. While perisynaptic neuronal neurotransmitter transporters contribute to uptake, astrocytes are also major mediators also, with transporters concentrated on processes highly proximal to the synaptic cleft, forming the so-called tripartite synapse. In the case of the major excitatory neurotransmitter glutamate, two transporters are expressed on astrocytes: EAAT1/GLAST (gene name *Slc1a3*) and EAAT2/GLT-1 (gene name *Slc1a2*). In *in vitro* monoculture, astrocytes express low levels of both transporters compared with *in vivo* [22–24] and consistent with this, functional uptake capacity measured by direct patch clamp analysis is also low [25]. Swanson and colleagues demonstrated that expression of both GLT-1 and GLAST are increased when astrocytes are cocultured with neurons [22], pointing to a neuron-derived signal being required to maintain GLT-1 and GLAST expression *in vivo*. Indeed, astrocytes acutely isolated from mouse brains show high levels of GLT-1 and GLAST, which decline rapidly when they are maintained away from their normal CNS microenvironment [25]. Moreover, GLT-1/GLAST expression can be driven back up by adding neurons to the culture [25], showing that the process is highly dynamic. One reason why astrocytes are particularly effective at clearing synaptic glutamate is that once taken up, glutamate is metabolised, primarily to either to glutamine (by the actions of glutamine synthetase, GLUL) or α-ketoglutarate (by the actions of glutamate dehydrogenase, GLUD1) [26]. This metabolism reduces the build-up of intracellular glutamate that would impair uptake by creating an unfavourable concentration gradient. This is an issue for neurons that have weak glutamate-metabolising pathways since expression levels of both GLUL and GLUD1 are low [23,26]. Of note, both Glul and Glud1 expression are low in astrocyte monocultures and are subject to the same neuron-dependent transcriptional induction as Slc1a2 and Slc1a3 (GLT-1 and GLAST) [25], meaning that astrocytic glutamate uptake and metabolism are coregulated. This makes biological sense as both components are important for efficient uptake.

Mechanistically, neuron-dependent induction of astrocyte glutamate transporters involves neuron-to-astrocyte Notch signalling [24]. The Notch signalling pathway is strongly active in astrocytes *in vivo* [23] but expression of classical Notch target genes *Hes5* and *Hey2* falls, once astrocytes are isolated away from their *in vivo* environment [25]. Neuronal coculture drives these genes back up, but blocking Notch signalling (with a gamma secretase inhibitor) prevents this, and prevents the induction of glutamate transporters at the mRNA and functional level [25]. Moreover, constitutive activation of the canonical Notch effector CBF1 is sufficient to elevate glutamate uptake capacity in the absence of neuron coculture. Note that there is no evidence that Slc1a2, Slc1a3, Glul, or Glud1 are direct Notch/CBF1 target genes; our working hypothesis is that they form a secondary response to the activation of the direct Notch/CBF1 target genes. In conclusion, while the Notch pathway plays an important role in committing neural progenitor cells towards an astrocytic fate [27], it also may play a role in maintaining key aspects of astrocytic function postdifferentiation. Of note, while the inhibition of Notch signalling blocked neuron-dependent induction of astrocytic glutamate uptake capacity in a coculture, it did not prevent neurons from promoting a stellate morphology (Philip Hasel and Giles Hardingham, unpublished observations), consistent with neurexin–neuroligin being the primary driver of this process [14].

Interestingly, endothelial cells are also capable of inducing GLT-1 in astrocytes in a Notch-dependent manner [28]. Thus suggests that brain endothelial cells as well as neurons might contribute to Notch-dependent control of astrocytic glutamate uptake *in vivo*, although the fact that astrocytic endfeet are thought to primarily contact the capillary basement membrane rather than endothelial cells directly may limit a role for endothelial-derived juxtacrine signalling. Neuron- and/or endothelial cell-specific deletion (ideally inducible) of highly expressed notch ligands, such as Jag2 and Dlk2 (in neurons) would enable the role of each cell type in activating astrocyte notch signalling *in vivo* to be assessed. Reduction in astrocytic GLT-1 and GLAST is seen in a variety of pathological situations, which are regarded as a potential marker of reactive astrocytes, or at least a subset of them [29]. In Alzheimer’s disease (AD) and other dementias, a reduction in astrocytic GLT-1 and GLAST is observed in animal models and postmortem tissue, and is also a feature of ageing, the strongest risk factor for AD and other neurodegenerative diseases [24,30]. It is currently unclear whether reduction in GLT-1 and GLAST is due to deficits in notch signalling (e.g., hypoexpression of neuronal notch ligands or astrocytic notch), a reduction in neuron–astrocyte contacts, or a different mechanism entirely. Nevertheless, such deficits will prolong postsynaptic exposure to glutamate, reducing the fidelity of synaptic transmission, as well as cause spillover onto extrasynaptic NMDARs, which couple preferentially to neurodestructive pathways [31,32]

Although less well-studied, it appears likely that neurons also regulate astrocytes’ capacity for the uptake and metabolism of GABA, the main inhibitory neurotransmitter in the CNS. Similar to GLT-1 and GLAST, the main astrocytic GABA transporters GAT-1 (Slc6a1) and GAT-3 (Slc6a11) are expressed weakly in monocultures compared with *in vivo* [23], and are robustly induced by neuronal coculture [25]. GABA metabolism begins with its conversion to succinate semialdehyde in a reaction catalysed by ABAT (4-Aminobutyrate Aminotransferase), which is also regulated in a similar way by neuronal coculture, though the Notch-dependency of this process has not been investigated. Nevertheless, it seems likely that the capacity of astrocytes for GABA- as well as glutamate-homeostasis relies in part on continuous instruction from neurons. Interestingly, peritumour astrocytes isolated from glioblastoma patients exhibit a marked lowering of expression of genes encoding GLT-1, GLAST, GAT-1, and GAT-3 of between 70 and 85% [33]. This dramatic loss could conceivably be due to a reduction in neurons in the proximity of the tumour and therefore reduced neuron–astrocyte contacts needed for notch activation.

## Regulation of K^+^ buffering

Astrocytes play an important role in CNS K^+^ homeostasis of which there are two types: net uptake and spatial buffering, both of which have a significant impact on neuronal excitability [34]. Net K^+^ uptake is predominantly mediated by the Na+/K+ transporting ATPase (NAK), a heterodimer of one α and one β subunit. The dominant subunits in astrocytes are the α-2 and β-2 (Atp1a2 and Atp1b2), but the high expression of the catalytic subunit Atp1a2 *in vivo* is not seen in astrocyte monocultures [23], yet can be rescued by coculture with cortical neurons in a manner analogous to the control of neurotransmitter uptake [25]. Aside from net K^+^ buffering, spatial buffering is also crucial function of astrocytes, allowing for local uptake in areas of high [K^+^] such as regions proximal to intense neuronal activity, and dissipation/redistribution through the gap junction-coupled astrocyte network [34]. Inwardly rectifying K^+^ channels (Kirs) are major mediators of spatial K^+^ buffering. In contrast with regulation of Atp1a2, the major Kir channel in astrocytes, Kir4.1 (gene name *Kcnj10*) is expressed at similar levels in monoculture compared with *in vivo* [23], and its expression is not influenced by coculture with cortical neurons [25]. As such, astrocytic spatial K+ buffering may not require continuous instruction from neurons or other contributors to the CNS microenvironment to be maintained. Nevertheless, astrocytic Kir4.1 expression is affected in disease: it is reduced in mouse models of ALS [35] as well as Huntington’s disease, which the authors directly linked to altered striatal neuronal excitability (due to elevated extracellular K^+^) and aspects of physical deterioration of the mice [36]. Furthermore, reduced astrocytic Kir4.1 expression causes impaired GLT-1-mediated glutamate uptake due to GLT-1 coupling glutamate uptake to K+ extrusion, and the whole process requiring a hyperpolarised membrane potential [37]. K+ levels are also dynamic regulators of astrocytic glucose metabolism [6]. Thus, a greater understanding of what regulates Kir4.1 expression in astrocytes may yield astrocyte-normalising strategies that alter disease trajectory.

## Regulation of glucose metabolism

Glucose metabolism follows two primary pathways: the glycolytic pathway and the pentose-phosphate pathway (PPP). Glycolysis serves primarily to generate ATP, both in its own right, as well as providing the end product pyruvate to enter mitochondria to be further oxidised through the tricarboxylic acid (TCA) cycle (after oxidation to acetyl-CoA) and provide NADH to drive oxidative phosphorylation. Pyruvate can also serve as an energy source for other cells to feed into their own TCA cycle by being reduced to lactate (by lactate dehydrogenase), exported (through monocarboxylic acid transporters, MCTs) and taken up by other cells (also by MCTs) and oxidised back to pyruvate. In contrast with the role of glycolysis in energy production, metabolism of glucose through the PPP functions primarily to generate NADPH, an essential cofactor for the regeneration of the reduced form of glutathione (GSH) from the oxidised form. Metabolic intermediates in both pathways feed into various biosynthetic processes (e.g., production of nucleotides, phospholipids, GSH) but in bioenergetic terms the balance of a cell’s utilisation of glycolysis versus PPP can determine the extent to which they rely on oxidative phosphorylation for ATP production. For example, astrocytes synthesise GSH *de novo* so have a relatively low need for the PPP, leading to high levels of glycolytic flux and ultimately production and release of lactate [1]. In contrast, neurons divert a large proportion of glucose through the PPP by targeting a positive regulator of glycolysis, PFKFB3, for continuous degradation [38]. Disruption of this degradation, which happens under excitotoxic conditions, leads to elevated glycolysis and reduced PPP activity, the latter reducing NADPH generation and causing oxidative stress [39]. As such, healthy neurons rely heavily on oxidative phosphorylation for ATP generation and take up astrocyte-released lactate to convert into pyruvate and feed into the TCA cycle (the so-called astrocyte-neuron lactate shuttle [40]). Early evidence that neurons may influence astrocyte metabolism (and therefore lactate export) came from combined FISH and immunohistochemistry study, which reported an elevated in genes involved in glycolysis and glycogen metabolism in astrocytes upon coculture with neurons [41].

We recently observed that the capacity of astrocytes to supply neurons with lactate is indeed subject to regulation by neurons. However, unlike neurotransmitter uptake or morphological transformation, this control was not exerted via contact-dependent juxtacrine signalling, but instead occurs in response to neuronal firing activity. It has been known for decades that neuronal activity triggers changes in gene expression in neurons [42–44]. However we observed that hundreds of genes in astrocytes are also subject to induction or repression in response to neuronal activity [25]. The Ca2+/cAMP-responsive transcription factor CREB was found to be a major mediator of activity-dependent gene induction in astrocytes, with cAMP-PKA signalling being the likely activating intracellular signal [25]. Among the genes induced in astrocytes by synaptic activity include a cluster associated with glycolysis composed of genes encoding transporters and enzymes responsible for each of the eleven steps from glucose uptake, glycolysis, pyruvate reduction to lactate and export from the cell, as well as the aforementioned glycolysis regulator *Pfkfb3* [25]. This co-ordinated up-regulation is associated with a CREB-dependent increase in astrocytic glucose metabolism, as well as elevated pyruvate production and lactate export [25]. Since neuronal synaptic activity is energetically expensive, it is tempting to speculate that this neuron-to-astrocyte signalling has evolved to tune the metabolic properties of astrocytes to meet the energy requirements of nearby neurons. Expression of most of these activity-dependent genes decline in the astrocytes of ageing humans [25,33]. More specifically, glucose metabolism is reduced in AD and in AD models, including in astrocytes [8,30,45], suggestive of either reduced neuronal activity, or weaker neuron-to-astrocyte signalling.

## Regulation of redox buffering and other stress responses

Astrocyte-mediated redox support is another important homeostatic role of astrocytes. Unlike neurons, astrocytes have the capacity to synthesise and store large amounts of GSH, which is released in response to oxidative stress through the multidrug-resistance protein MRP1. It can act directly in the extracellular space to promote redox balance, for example, in detoxifying peroxides. However it can also be broken down to provide precursors for neurons to produce their own GSH [46–48]. Genes whose products are involved in GSH biosynthesis (*Gclc, Gclm, Gss*) and export (Abcc1/MRP1) are elevated in astrocytes in comparison to neurons, but expression is stable when astrocytes are isolated away from the brain and not influenced by neuronal coculture [23,25].

Neuronal activity is known to stimulate neurons’ own intrinsic antioxidant defences, via the transcriptional up-regulation of systems centred on thioredoxins/peroxiredoxins as well as GSH synthesis and recycling, potentially a response to elevated levels of metabolic activity-associated reactive oxygen species production [43,49–53]. However, neuronal activity does not influence expression of these genes in astrocytes. Two antioxidant genes are notably subject to regulation by neuronal activity in astrocytes: the extracellular GSH peroxidase GPX3 and the extracellular superoxide dismutase SOD3 [25]. Thus, the extent of influence of neuronal activity on redox systems may extend to intraneuronal and extracellular compartments, but not astrocytes themselves.

This absence of neuronal control is perhaps not surprising when one considers that many antioxidant and detoxification and proteostasis genes, including those listed above, are regulated by the master transcriptional regulator Nrf2 (*Nfe2l2*), expressed at high levels in astrocytes but silenced in neurons by epigenetic and post-translational mechanisms [54,55]. High Nrf2 expression is another stable feature of astrocytes not requiring any noncell-autonomous instruction [23,25], so it is perhaps expected that basal expression of Nrf2 target genes is similarly neuron-independent. However, Nrf2 is itself activated by stressors such as mild oxidative insults that inhibit its ubiquitin-mediated degradation [56]. As such Nrf2-dependent phenotypes such as the capacity of astrocytes to neutralise reactive oxygen species are dynamically regulated even when expression of Nrf2 mRNA itself is unchanged. For example, Nrf2 activation by brief ischemia promotes neuroprotection [57–59]. Moreover, endogenous Nrf2 activation in astrocytes is an adaptive-protective response to neuron-derived Aß and tau pathology, and driving Nrf2 expression in astrocytes is sufficient to reduce pathology and slow disease progression in models of Aß and tau pathology [60].

## Caveats and future investigations

It is important to note that for all findings based on transcriptomic data that these studies simply provide hypotheses as to the potential functional changes effected by neuronal signals. Such hypotheses should ideally be tested by studying protein expression and functional assays such as glutamate uptake, glucose utilisation, lactate export, and GSH production. Moreover, findings should be ideally confirmed in human astrocytes since signal-dependent gene expression is subject to a degree of evolutionary divergence in neurons [44,61–63], and so is likely to be the case in astrocytes as well. While primary foetal and iPSC-derived astrocytes provide a valuable resource, the use of acutely isolated human adult astrocytes from surgically resected tissue (approximately nonpathological, such as tissue distal from a deep-lying glioblastoma) may represent the ‘gold standard’ for studying neuron-to-astrocyte communication, as well as providing a valuable comparator for assessing the similarities (and differences) from ‘control’ postmortem tissue, with regard to both tissue slice spatial ‘omics, as well as single cell/single nucleus’ omics.

## Future perspectives and concluding remarks

**Figure 1:**
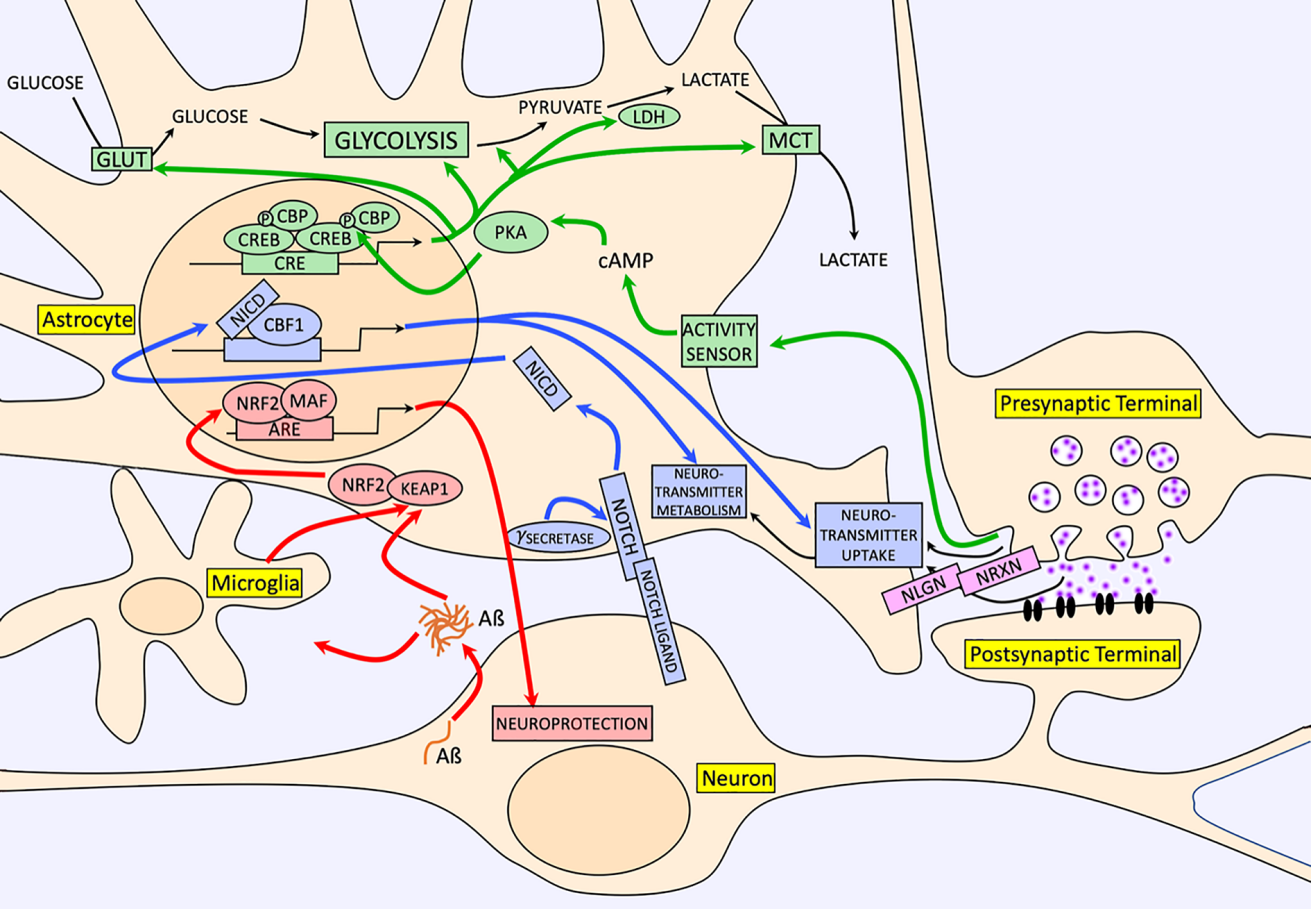
A selection of neuron-derived signals that induce long-lasting neurosupportive functions in astrocytes The green arrows and proteins/processes illustrate the impact of neuronal activity on astrocytic glucose uptake, glycolysis, and lactate export via the cAMP/PKA-dependent activation of CREB-dependent genes in the aforementioned pathway [25]. Note that the precise signal (and therefore receptor) released by active neurons awaits discovery. The pink proteins highlight the role of astrocytic neuroligin–neurexin signalling in both driving morphological complexity and excitatory synapse formation [14]. The blue arrows and proteins/processes illustrate the impact of neuron-to-astrocyte notch signalling on expression and functional activity of neurotransmitter uptake [25]. The red arrows and proteins/processes illustrate the adaptive-protective response of astrocytes to Aß pathology [60].

Figure 1 gives a summary of the neuron-derived signals, discussed in the present review that impacts on astrocyte function. Why so many? It makes teleological sense for astrocyte phenotypes to be subject to regulation by neurons, since in theory, it enables neurons to tune the properties of nearby astrocytes to their needs. However, there still remains much to understand about the extent of this control. Over 2000 genes in astrocytes are induced or repressed by neurons [25], and the functional consequences of these changes is only known for a handful of genes. The relative contribution of Notch versus Neuroligin in mediating these changes is also currently unclear, and it may be that other signalling pathways are involved too. The same knowledge gap exists for astrocytic genes induced or repressed by neuronal activity in that there is much to learn about the functional consequences of most of the transcriptional changes seen. Moreover, the nature of the activity-dependent intercellular signal responsible for inducing these changes awaits discovery. Nevertheless, it is clear that the capacity of astrocytes to perform various homeostatic functions is not fixed but subject to regulation by physiological signalling from neurons. This type of phenotypic plasticity should be viewed as different from the changes that take place in ‘reactive astrocytes’ where changes to place in response to pathological situations. Overall, the knowledge that a wide range of astrocytic neurosupportive functions can be controlled raises the possibility of manipulating them artificially for therapeutic benefit in chronic brain disorders.

## Summary

- Astrocytes play key neuro-supportive roles in the healthy brain, including multiple aspects of metabolic homeostasis.
- While astrocytes are known to change their phenotype in disease (acquiring ‘reactive’ or atrophic states, whether astrocyte properties are modifiable or remain fixed in a healthy brain is not well understood.
- Recent work shows that neurons signal to astrocytes to modify multiple aspects of their neurosupportive phenotype in development and maturity.
- Neurons exert their effects by a combination of activity-dependent and contact-dependent signalling.
- Many neuron-regulated signals direct astrocytes to boost their neurosupportive/homeostatic functions, and many of these functions decline in age-related brain disorders like AD.
- Mechanistic insight into these pathways shows how astrocyte neurosupportive/homeostatic properties may be manipulated to generate a desirable phenotype that might slow the trajectory of brain disorders.

## Open Access

Open access for the present article was enabled by the participation of The University of Edinburgh in an all-inclusive *Read & Publish* agreement with Portland Press and the Biochemical Society under a transformative agreement with JISC.

## Abbreviations

ABAT: 4-aminobutyrate aminotransferase
AD: Alzheimer’s disease
ALS: Amyotrophic lateral sclerosis
GABA: Gamma-Aminobutyric Acid
GLUD: glutamate dehydrogenase
GLUL: glutamine synthetase
GSH: glutathione
Kir: inwardly rectifying K+ channel
MCT: monocarboxylic acid transporter
MRP: multidrug-resistance protein
NAK: Na+/K+ transporting ATPase
NMDAR: N-methyl-D-aspartate receptor
NO: nitric oxide
PPP: pentose-phosphate pathway
TCA: tricarboxylic acid
